# MOREOVER: multiomics MR-guided radiotherapy optimization in locally advanced rectal cancer

**DOI:** 10.1186/s13014-024-02492-9

**Published:** 2024-07-25

**Authors:** Luca Boldrini, Giuditta Chiloiro, Silvia Di Franco, Angela Romano, Lana Smiljanic, Elena Huong Tran, Francesco Bono, Diepriye Charles Davies, Loris Lopetuso, Maria De Bonis, Angelo Minucci, Luciano Giacò, Davide Cusumano, Lorenzo Placidi, Diana Giannarelli, Evis Sala, Maria Antonietta Gambacorta

**Affiliations:** 1 Gemelli Advanced Radiotherapy center, Fondazione Policlinico Universitario “A. Gemelli” IRCCS, Rome, Italy; 2 Radiomics GSTeP core research facility, Fondazione Policlinico Universitario “A. Gemelli” IRCCS, Rome, Italy; 3 Medicina Interna e Gastroenterologia, CEMAD Centro Malattie dell’Apparato Digerente, Dipartimento di Scienze Mediche e Chirurgiche, Fondazione Policlinico Universitario “A. Gemelli” IRCCS, Rome, Italy; 4 Department of Medicine and Ageing Sciences, ”G. d’Annunzio” University of Chieti-Pescara, Chieti, Italy; 5 Genomics GSTeP core research facility, Fondazione Policlinico Universitario “A. Gemelli” IRCCS, Rome, Italy; 6 Bioinformatics GSTeP core research facility, Fondazione Policlinico Universitario “A. Gemelli” IRCCS, Rome, Italy; 7 Medical Physics unit, Mater Olbia Hospital, Olbia, Italy; 8 Medical Physics unit, Fondazione Policlinico Universitario “A. Gemelli” IRCCS, Rome, Italy; 9 Biostatistics Unit, Fondazione Policlinico Universitario “A. Gemelli” IRCCS, Rome, Italy; 10 Gemelli Advanced Radiology center, Fondazione Policlinico Universitario “A. Gemelli” IRCCS, Rome, Italy; 11 Istituto di Radiologia, Università Cattolica del Sacro Cuore, Rome, Italy

**Keywords:** Rectal cancer, Magnetic resonance guided Radiation Therapy, Radiomics, Gut microbioma, Circulating tumor DNA

## Abstract

**Background:**

Complete response prediction in locally advanced rectal cancer (LARC) patients is generally focused on the radiomics analysis of staging MRI. Until now, omics information extracted from gut microbiota and circulating tumor DNA (ctDNA) have not been integrated in composite biomarkers-based models, thereby omitting valuable information from the decision-making process. In this study, we aim to integrate radiomics with gut microbiota and ctDNA-based genomics tracking during neoadjuvant chemoradiotherapy (nCRT).

**Methods:**

The main hypothesis of the MOREOVER study is that the incorporation of composite biomarkers with radiomics-based models used in the THUNDER-2 trial will improve the pathological complete response (pCR) predictive power of such models, paving the way for more accurate and comprehensive personalized treatment approaches. This is due to the inclusion of actionable omics variables that may disclose previously unknown correlations with radiomics. Aims of this study are: - to generate longitudinal microbiome data linked to disease resistance to nCRT and postulate future therapeutic strategies in terms of both type of treatment and timing, such as fecal microbiota transplant in non-responding patients. - to describe the genomics pattern and ctDNA data evolution throughout the nCRT treatment in order to support the prediction outcome and identify new risk-category stratification agents. - to mine and combine collected data through integrated multi-omics approaches (radiomics, metagenomics, metabolomics, metatranscriptomics, human genomics, ctDNA) in order to increase the performance of the radiomics-based response predictive model for LARC patients undergoing nCRT on MR-Linac.

**Experimental design:**

The objective of the MOREOVER project is to enrich the phase II THUNDER-2 trial (NCT04815694) with gut microbiota and ctDNA omics information, by exploring the possibility to enhance predictive performance of the developed model. Longitudinal ctDNA genomics, microbiome and genomics data will be analyzed on 7 timepoints: prior to nCRT, during nCRT on a weekly basis and prior to surgery. Specific modelling will be performed for data harvested, according to the TRIPOD statements.

**Discussion:**

We expect to find differences in fecal microbiome, ctDNA and radiomics profiles between the two groups of patients (pCR and not pCR). In addition, we expect to find a variability in the stability of the considered omics features over time. The identified profiles will be inserted into dedicated modelling solutions to set up a multiomics decision support system able to achieve personalized treatments.

## Background

The current approach to predicting the achievement of pathological complete response (pCR) in patients affected by locally advanced rectal cancer (LARC) focuses mainly on magnetic resonance imaging (MRI) derived radiomics [1–5]. Several models have revealed the predictive value of images acquired before, during or after the end of chemoradiotherapy (CRT), supporting the possibility of identifying image-based biomarkers of response and modulating treatment accordingly [6].

The ability to predict which patients will achieve pCR is paramount for the customization of multidisciplinary oncological care.

This approach allows for personalized treatments for patients considered as “non-responders” to standard neoadjuvant chemoradiotherapy treatments (nCRT), while guiding those predicted to be “responders” toward more conservative therapeutic approaches aimed at organ preservation [2]. Indeed, local excision (LE) and “Watch and Wait” (W&W) strategies are now the most common approaches to reduce morbidity and toxicity associated with surgical overtreatments, such as total mesorectal excision (TME) following successful nCRT [3, 4].

The advent of magnetic resonance imaging guided radiotherapy (MRIgRT) systems caused a paradigm shift in this field, providing in depth imaging details for image guided radiotherapy (IGRT) purposes and the possibility to perform online adapted radiotherapy plans. However, the full potential of incorporating such imaging data into predictive models is still to be thoroughly investigated.

One of the most promising pilot studies is the “THeragnostic Utilities for Neoplastic DisEases of the Rectum” (THUNDER-2) study, which recently completed its enrolment phase [7]. The THUNDER-2 trial is a prospective, interventional, not-for-profit phase 2 trial (NCT04815694) designed to increase pCR rates by 10% in patients with LARC (cT2-4cN0-2cM0). These patients receive nCRT on a 0.35 T MR-Linac system and undergo a risk stratification through a decision support system, dependent on the radiomic analysis of 0.35T MR images.

This approach is based on the Early Regression Index (ERI) [9] and appears to be very effective in predicting pCR (with an AUC of 0.93 when applied on the 10th nCRT fraction), allowing intensification of treatment only in patients who do not respond to initial therapy. This treatment intensification is delivered through an online adaptive dose escalation to the residual gross tumor volume (GTV), achieving a biologically effective dose (BED) of at least 74.6 Gy.

Despite being very effective, the current models do not take into account essential omics data from other sources, such as the gut microbiota and circulating tumour DNA (ctDNA), which may disclose crucial information for a more comprehensive decision-making process.

In particular CtDNA, a promising prognostic indicator and certain microbial taxa, such as Thermi, have already been identified in LARC patients who have obtained pCR, which could be suggestive of their roles in improving selection processes [10–12][13,14].

The MOREOVER project aims to build on the existing evidence derived from the THUNDER-2 study with gut microbiota and ctDNA omics information, by exploring the possibility to enhance the predictive performance of a model based on composite biomarkers in order to achieve better personalize treatments, improve outcomes and reduce toxicity rates.

## Methods/design

### Study design

This is a single center prospective clinical trial which comprises three consecutive phases.

In the phase one, we aim to investigate predictive biomarkers for pCR in three omics domains: radiomics, microbiome, and ctDNA. This phase involves the enrolment of 116 patients over two years [7].

The enrolment in the MOREOVER study will be conducted as part of an observational amendment to the ongoing THUNDER-2 trial, adhering to its original inclusion criteria [7].

Clinical data from patients with locally advanced rectal cancer, who are undergoing nCRT will be prospectively collected utilizing the institutional RedCap application to minimize data loss or lack of inter-actionability.

Longitudinal pCR predictive models based on microbiome and ctDNA alone and derived mixed models will be set up and applied in the subsequent study phase.

In phase 2, a prospective interventional study involving 165 patients will be conducted, using an online MRIgRT adaptive boost for patients predicted to be non-responders. The aim is to increase pCR rates to 40%, exceeding the current average of 25%.

**Fig. 1:**
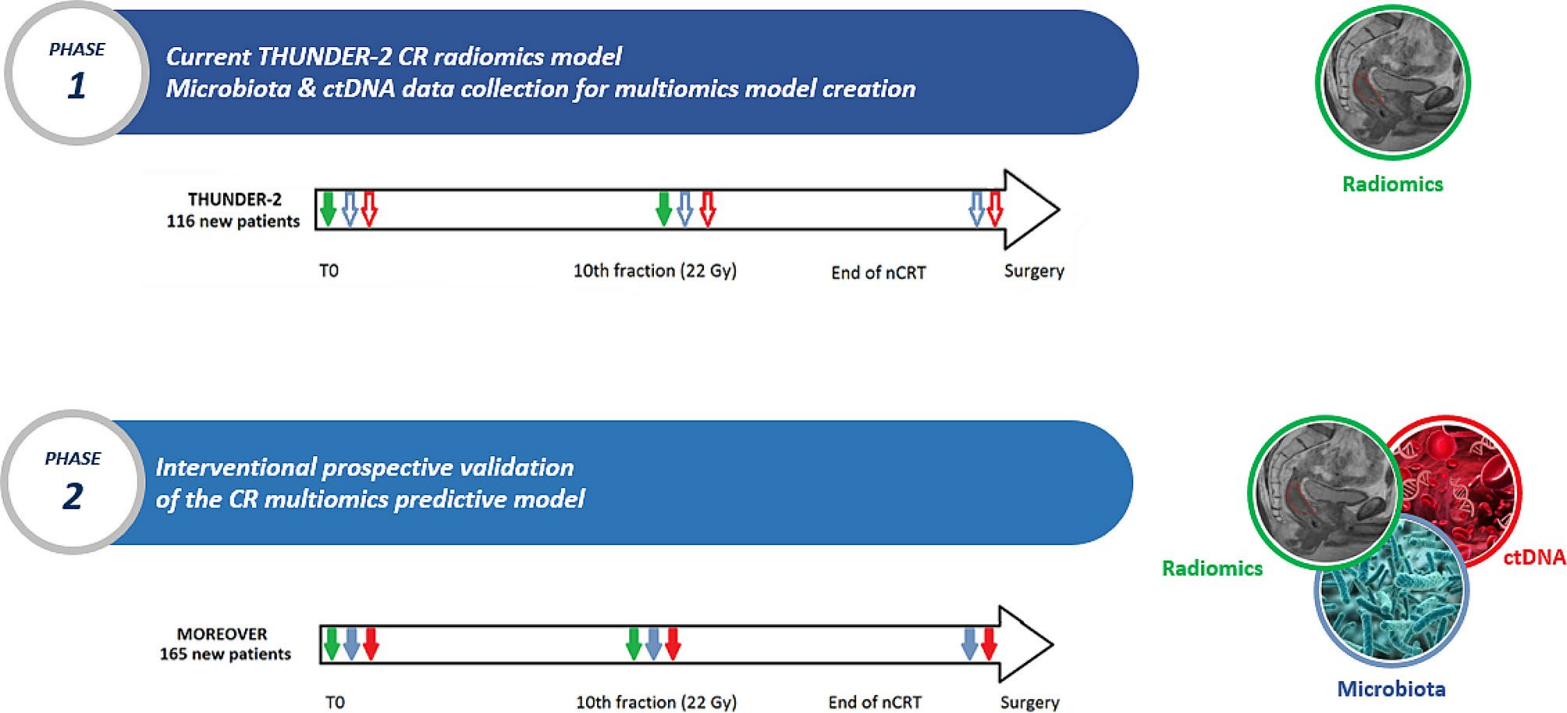
Data Collection Timeline. The three critical data collection time points are reported: (1) Prior to neoadjuvant chemoradiotherapy (nCRT), (2) During nCRT (10th fraction), and (3) Prior to surgery. CR: complete response

Figure 1 summarizes the two phases.

The study will integrate several innovative therapies for non-responder patients, including radiomics-guided dose escalation of radiotherapy with online adaptive MRI optimisation, genomics-adapted radiotherapy prescriptions and microbioma-based interventions (i.e. microbioma transplant).

### Study objectives

The primary objective of the MOREOVER study is to develop an innovative multi-omics model that integrates a diverse range of biomarkers (radiomics, microbiome and ctDNA) to improve the personalisation of nCRT in LARC patients aiming to an increased pCR rate.

### Ethical considerations and data management

This study has been approved by the Ethics Committee of the Fondazione Policlinico Universitario “A. Gemelli” IRCCS (ID: 3460) as a substantial amendment of the THUNDER-2 trial [7].

The MOREOVER study is totally funded by the Associazione Italiana per la Ricerca sul Cancro (AIRC) - Next Generation Clinician Scientist Call grant, assigned to the principal investigator in the 2022 edition (ID 28,614).

This research adheres to the latest iteration of the Declaration of Helsinki and complies with Italian laws and regulations. Any modifications to the protocol that could impact the study’s execution, patient welfare or safety, such as changes to objectives, design, participant criteria, sample size, procedures, or significant administrative elements, were included in a formal protocol amendment approved by the Ethics Committee.

The protocol was realized in line with good clinical practice (GCP) principles. Prior to trial inclusion, patients will receive comprehensive explanations regarding the rationale, benefits, and potential side effects before voluntarily consenting to participate. Each patient or patient representative will formally sign detailed informed consent form. No interventions will be administered before informed consent is obtained. The involved medical team will handle unexpected serious adverse events, which will be thoroughly documented by the study coordinator. All study-related information will be collected and securely stored according to GDPR principles.

### Sample size

The first phase of the MOREOVER study aims to improve the AUC of the THUNDER-2 study [7] by incorporating omics elements into the original radiomics-only model. The goal is to develop a predictive model for pCR that excludes AUC values below 0.82, which is the lower limit of the 95% confidence interval of the previous model. To generate longitudinal microbiome data linked to disease resistance and to describe genomic patterns and evolution of ctDNA data during nCRT, we plan to apply non-parametric statistical analysis based on binary outcomes. To ensure that binary variables derived from dynamic microbiota and ctDNA analysis fit pCR results in the two patient subgroups, we observed a 29% difference in the frequency of each variable with a variable increase in pCR rate (from 36 to 65%). Assuming the same number of cases in each subgroup, with an alpha error of 0.05 and power (1 - beta) of 0.8, we determined the necessary sample size using Fisher’s exact test to be 116 patients.

In the second phase, a non-inferiority approach will be used to integrate multi-omics data into a single comprehensive decisional support system. This system will be used to improve the predictive performance of the current model, which is solely based on radiomics. We estimate that an AUC of 0.95 for the new test, a sample size of 96 patients will be sufficient to exclude AUC values below 0.83. Taking into account a dropout rate of 10%, the total number of patients for the MOREOVER trial is set at 165 [15]. All analyses will be conducted using the R software environment (R Foundation for Statistical Computing, Vienna, Austria) and the Python framework.

### Radiotherapy setting

The study will make use of a 0.35 T MR Linac (MRIdian, ViewRay Systems Inc) for treatment delivery, with patients immobilized in a supine position using the Fluxboard device (FluxboardTM, MacroMedics, The Netherlands).

0.35 T MR images will be obtained at simulation and daily throughout MRIgRT treatment of a total number of 26 image sets per patient.

The Early Regression Index (ERI) will be calculated at the 10th fraction, as extensively described in [7].

The Gross Tumor Volume (GTV) will be delineated on simulation MRI at the 10th fraction. MRI-based ERI calculated at this point will result in the adaptation of treatment plans. For ERI < 13.1, patients will continue their original treatment with a total dose of 55 Gy to the Planning Target Volume 2 (PTV2), while for ERI > 13.1, the treatment plan will be personalized, focusing on GTV3 (identified at the 10th fraction) as a new therapy volume. In such cases, the dose will be increased to 60.1 Gy (BEDα/β10 = 74.6 Gy) on PTV3, utilizing an online adaptive approach for real-time optimization [16].

The CTV1 which includes the primary rectal tumor, total mesorectum and selected lymphatic drainage stations, will be delineated manually according to the guidelines proposed by Valentini et al. [17] and the PTV1 will correspond to the CTV1 + 0.5 cm in all directions.

The CTV2 will include the primary rectal tumour plus the corresponding mesorectum, and PTV2 will be the CTV2 + 0.5 cm in all directions.

In case of the need for treatment intensification, PTV3 will be defined as the GTV delineated on MRI acquired at the fraction 10 (at 22 Gy) with an added isotropic margin of 0.3 cm.

The treatment comprises 25 long-course radiotherapy fractions with a total prescription dose of 55 Gy in fractions of 2.2 Gy to PTV2 and 45 Gy in fractions of 1.8 Gy to PTV1, following a simultaneous integrated boost (SIB) protocol [8].

Intensity Modulated Radiation Therapy (IMRT) with an “inverse planning” approach will be used for dose optimization and the prescription dose will be normalized to the target mean according to ICRU 83 [18].

### Radiomics analysis

The radiomics analysis will follow the approach used in the THUNDER-2 trial.

Imaging data will consist of 0.35T MR images and the corresponding GTV contours. The data will be stored in DICOM format and the region of interest (ROI) will be segmented in RTSTRUCT.RS format. Radiomics feature extraction and computation will strictly adhere to the IBSI ontology for histogram, morphological, fractal and texture features. The MODDICOM platform developed by our Radiomics Core Research Facility will be used for feature extraction [19]. Radiomics features will be automatically stored in .csv files. This approach will ensure a robust and stable radiomics analysis pipeline, reducing the risk of unintended model changes or deviations in the MOREOVER study.

### Microbiome data collection

Gut microbial metagenomics, metabolomics and metatranscriptomics will be performed on stool samples collected from patients at three different time-points: before nCRT, upon reaching a dose of 22 Gy and within one week after restaging MRI.

After collection, all samples will be stored at -80 °C. Shotgun metagenomics will be performed on the Illumina NovaSeq platform [20]. We will generate > 7.5Gb/sample of 150nt paired end reads (insert size ~ 150nt). This will be sufficient to cover a 2 × 4 Mb bacterial genome present at an abundance of 0.1% after accounting for human DNA reads removal, and to detect organisms at abundances as low as 0.01% with our marker-based strategy.

The taxonomical and functional profiles of each sample will be analyzed using MetaPhlan3 and Humann3 pipelines, respectively. To explore the functional potential of the gut microbiome, the sequenced metagenomes will be analyzed by combining two approaches: de novo assembling using available software (e.g. Meta-Velvet) and ORF annotation using well-established web annotation servers (e.g. MG-RAST and IMG/M).

Analyses will be carried out to define taxonomic and functional differences in the microbiome. Statistical analysis will be performed using QIIME and GraphPad Prism 6 through non-parametric tests. The correlation between the gut microbiota structure and clinical data will be assessed using correlation (e.g. Spearman and Kendall tau) tests and quantile regression tests, adjusted for demographic data.

We will capitalize on our laboratory’s extensive background and expertise in metagenomic data pre-processing: quality control, assembly, normalization/rarefaction, phylogenetic annotation, metagenomic species reconstruction, functional mapping to the latest gut microbiome gene catalogue, and microbial trait analyses. For metatranscriptomics, sample preparation will use the most recent and validated protocol for mRNA fraction enrichment by rRNA depletion as per the Illumina protocols. cDNA libraries will be synthetized and the pipeline described above will follow.

### Blood markers and circulating tumor DNA (ctDNA)

Blood-based biomarkers describing cancer-related features and the host immune environment (i.e. neutrophil/lymphocyte ratio, CEA, CA 19.9) will be extracted from standard blood samples taken before the start of nCRT, when a dose of 22 Gy is reached and within one week of restaging MRI. The primary objective is to support outcome prediction and identify new risk category stratification tools by describing the genomic pattern and ctDNA data evolution throughout nCRT treatment. Blood samples will be collected in Streck BCT tubes and processed within 2 h of plasma collection. The plasma will then be aliquoted into cryovials and snap frozen at -80 °C until ctDNA extraction. Plasma ctDNA will be isolated using QIAcube Connect kits according to the manufacturer’s protocols and Good laboratory practice (GLP). The Illumina TSO500 HT kit will be used for library preparation for targeted sequencing of ctDNA. 20–40 ng of DNA will be used as input for library preparation and sequencing (2 × 150 PE) will be performed on NovaSeq6000 (Illumina), aiming for at least 600 × average sequencing depth. The laboratory personnel will ensure appropriate ctDNA concentrations, as amounts less than 20 ng will not be processed. The sequencing library will be constructed using comprehensive genomic profiling. Raw sequencing data from the TSO500 HT analysis of ctDNA will be processed using the TSO500 associated software (DRAGEN TSO500 ctDNA 2.1.1) provided by Illumina on ICA platform. The ICA platfrom will be connected to the Clinical Genomic Workspace (CGW; Velsera) data portal to identify the following somatic variants: (1) pathogenic in the patient’s tumour type, (2) pathogenic in other tumour types, (3) variants reported in cancer, (4) variants of unknown significance, (5) polymorphisms.

### Predictive model development

Advanced machine learning techniques and deep learning algorithms will be employed to set up a predictive model for pCR in LARC patients, which will include data from radiomics, microbiome, and ctDNA analyses. Reicever operating characteristic (ROC) curve will be calculated for each model and the corresponding area under the curve (AUC) value will be determined using the DeLong method for the quantification of the 95% confidence interval. The Youden index will also be calculated at different threshold levels and the value showing the highest J index will be chosen as optimal cut-off: the accuracy, sensitivity and specificity will then be calculated accordingly. Appropriate statistical tools will be applied in order to reduce the risk of overfitting.

The developed biomarker-based models will be merged in a single matrix model, supporting the set-up of innovative therapeutic approaches in patients predicted as “non responders”. Examples include omics-driven radiotherapy dose escalation with online adaptive MR guided optimization (i.e. TCP-NTCP modeling with Poisson statistics dose-response relationships), genomics adjusted radiotherapy prescription and microbioma transplant or other support therapies, suggested by specific blood markers profiles.

## Discussion

The MOREOVER project aims to augment the current trial by incorporating gut microbiota and circulating tumor DNA (ctDNA) omics data. This approach seeks to enhance the predictive capability of a model reliant on composite biomarkers, thereby advancing personalized treatments, improving outcomes, and mitigating toxicity rates.

The MOREOVER study presents a significant opportunity to refine the characterization of rectal cancer through extensive genomics sequencing and microbiota analysis. This not only fosters innovative ideas, but also introduces new strategies for patient selection, toxicity mitigation, and risk stratification.

A dependable composite omics-based decision support system designed to predict response, has the potential to positively impact the Italian healthcare system. It can effectively personalize cancer treatments, thereby reducing the clinical, social, and economic burdens associated with overtreatments, such as surgery in completely responding patients. Additionally, it enables response-enhancing strategies like microbiota transplants, radiation therapy boosts, or genomic-adjusted radiotherapy prescriptions.

These anticipated benefits aimed at enhancing patients’ quality of life and overall management, align with the projected five-year timeline. This timeline allows for the initiation of prospective interventional trials using the composite biomarkers identified within the first two years of the project, for the successful implementation of the original radiomics model.

Beyond the specific project requirements, this unique dataset will serve as a foundation for further omics investigations and modelling for other outcomes, such as relapse or metastasis-free survival. It will also serve as a model for similar applications for other tumor types.

## Data Availability

No datasets were generated or analysed during the current study.

## Abbreviations

AIRC: associazione italiana per la ricerca sul cancro
AUC: Area under curve
BED: biologically effective dose
CR: Complete response
CRT: Chemoradiation therapy
ctDNA: circulating tumor DNA
CTV: Clinical target volume
DICOM: Digital imaging and communications in medicine
ERI: Early regression index
GPC: good clinical practice
GTV: Gross tumor volume
ICRU: International commission on radiation units and measurements
IGRT: image guided radiotherapy
IMRT: Intensity modulated radiation therapy
LARC: Locally andvanced rectal cancer
LE: Local excision
LINAC: Linear accelerator
MR: Magnetic resonance
MRgRT: Magnetic resonance guided radiation therapy
MRI: Magnetic resonance imaging
nCRT: Neoadjuvant chemoradiation therapy
NTCP: normal tissue complication probability
pCR: Pathological complete response
PTV: Planning target volume
RedCap: research Eletronic Data Capture
ROC: Receiver operating characteristic
SIB: Simultaneous integrated boost
TCP: tumor control probability
TME: total mesorectal excision
W&W: Watch and wait
